# Interplay of Receptor Status, Age, and Stage in Breast Cancer: A Prospective Analysis

**DOI:** 10.7759/cureus.85925

**Published:** 2025-06-13

**Authors:** Mariam Malik, Zeeshan R Mirza, Rana Bilal Idrees, Saba Nawaz, Jawairia Arif, Barira Ahmad, Summaya S Chaudry, Muhammad Hamid Chaudhary

**Affiliations:** 1 Radiology, Atomic Energy Cancer Hospital, Nuclear Medicine, Oncology and Radiotherapy Institute (NORI), Islamabad, PAK; 2 Diagnostic Radiology, Institute of Nuclear Medicine and Oncology Lahore (INMOL) Cancer Hospital, Lahore, PAK; 3 Radiology, Institute of Nuclear Medicine and Oncology Lahore (INMOL) Cancer Hospital, Lahore, PAK; 4 Histopathology, Shaheed Zulfiqar Ali Bhutto Medical University/Pakistan Institute of Medical Sciences (PIMS), Islamabad, PAK; 5 Cardiac Surgery, Chaudhry Pervaiz Elahi Institute of Cardiology, Multan, PAK

**Keywords:** breast carcinoma in situ, estrogen receptor (er) positive, her 2 negative, her-2-neu, metastatic lobular breast carcinoma

## Abstract

Background

Breast cancer is the most prevalent cancer among women globally, with significant variations in incidence and characteristics across different age groups and regions. Understanding the relationship between age, hormone receptor status, and breast cancer stage is crucial for developing effective treatment strategies.

Objectives

This study aimed to: (1) categorize the relationship between receptor status and the stage of breast cancer, (2) determine the frequency of different receptor statuses according to patient age, and (3) correlate the relationship between age and the stage of breast cancer among Pakistani women.

Materials and methods

A prospective analysis was conducted on 1003 breast cancer patients from a semi-government-run hospital in Lahore, Pakistan, between October 2021 to October 2023 using systematic sampling to recruit every fourth patient of breast cancer. Data on age, tumor grade, histopathological subtype, and hormone receptor status (estrogen receptor (ER), progesterone receptor (PR), human epidermal growth factor receptor 2/NEU (HER2/NEU), Ki-67) were collected and analyzed using SPSS v26 (IBM Corp., Armonk, USA). Chi-squared test was employed to explore associations between age groups and receptor status.

Results

The mean age of patients was 50.5 years, with a concentration of cases between 41 and 60 years. Invasive ductal carcinoma (IDC) was the most common subtype (91%). Grade II tumors were most prevalent (50.6%), followed by Grade III (45.5%). The majority of patients were ER-positive (62.4%), followed by PR-positive (52.3%), while HER2/NEU positivity was 44%. The most common receptor status was HER2/NEU negative and ER/PR positive (28.9%). Receptor status distribution varied significantly among age groups (p < 0.000), with younger patients more likely to have triple-negative breast cancers and older patients more likely to have ER/PR-positive, HER2/NEU-negative cancers. Ki-67 levels were assessed in 41.5% of patients, with higher levels observed in younger patients. Younger patients (20-30 years) had a higher prevalence of Grade III tumors, whereas older patients (over 60 years) more frequently had Grade II tumors. There were no significant differences in hormone receptor status distribution across cancer stages (p = 0.76). The stage of carcinoma did not significantly differ across age groups (p = 0.05).

Conclusion

The study highlights significant age-related differences in breast cancer pathology, particularly in tumor grade and receptor status, underscoring the need for age-specific treatment strategies. Younger patients tend to present with more aggressive tumors, necessitating tailored therapeutic approaches to improve outcomes.

## Introduction

Breast cancer is the most common cancer among women worldwide, affecting one in eight women during their lifetime [1]. It represents 16% of all female cancers and 25% of all invasive cancers in women [2]. In 2020, the WHO reported about 2.3 million new cases and 685,000 deaths from breast cancer [3]. The average age of diagnosis is 63 years in Western countries, but is younger in Asian and Arab countries, with averages of 48.8 years in Saudi Arabia, 51.92 years in South India, and 47.5 years in Pakistan [4, 5]. In Pakistan, one in nine women risks developing breast cancer, the highest incidence in Asia [6].

Younger women often develop more aggressive and advanced breast cancer, characterized by negative hormone receptor status, higher grades, and poor differentiation [7, 8]. Additionally, younger age is often linked to overexpression of human epidermal growth factor receptor 2/NEU (HER2/NEU), a biomarker associated with poorer prognosis and treatment challenges [9]. This highlights the need for tailored diagnostic and therapeutic strategies for younger patients.

Breast cancer treatment strategies consider various factors like age, tumor size, menopausal status, axillary nodal status, HER2/NEU expression, and hormone receptor status. Hormone receptor status, particularly estrogen receptor (ER) and progesterone receptor (PR), significantly influences treatment decisions and outcomes. ER- and/or PR-positive cancers respond better to hormonal therapies, while triple-negative cancers often require more aggressive treatments due to a lack of targeted therapies and poorer outcomes.

Breast cancer staging and grading are crucial for prognosis and treatment planning, with stages ranging from 0 (non-invasive) to IV (distant metastasis). However, the relationship between hormone receptor status and age among Pakistani women, especially from low-income and low-education backgrounds, is not well-explored. Socioeconomic barriers can delay diagnosis and treatment, affecting cancer stage and prognosis.

Khan et al. found a significant association between hormone receptor status and local ethnic groups in Pakistan, suggesting ethnicity may influence breast cancer biology and progression [10]. Despite these findings, more local research is needed to explore the age-hormone receptor status relationship in Pakistani women. Understanding this is crucial for developing age-specific screening and personalized treatment protocols.

This study aims to: 1) categorize the relationship between receptor status and breast cancer stage; 2) determine the frequency of different receptor statuses by patient age; and 3) correlate age and breast cancer stage.

The research seeks to provide insights into the interplay between age, receptor status, and breast cancer stage, informing clinical practices and public health policies. The findings could lead to more effective, personalized treatments, improving survival rates and quality of life for breast cancer patients in Pakistan and similar settings.

## Materials and methods

A prospective cross-sectional study was conducted at the Institute of Nuclear Medicine and Oncology Lahore, Atomic Energy Cancer Hospital (INMOL-AECH), Lahore, Pakistan, from 1st October 2021 to 30th October 2023. The study was designed following the guidelines of the International Council for Harmonisation of Technical Requirements for Pharmaceuticals for Human Use (ICH) and Good Clinical Practice (GCP) guidelines, and the ethical standards on human experimentation in our institution. The study was approved by the Institutional Ethics Committee on Human Investigation before the start (INMOL-AECH, Lahore, IRB# INMOL -53(40)), and written informed consent was obtained from all the patients. The participation of all participants was confidential and voluntary at all times. 

A systematic sampling technique was used to recruit participants in the study, with every fourth patient of breast cancer selected. All the patients fulfilling the inclusion criteria and voluntarily willing to participate were studied. Inclusion criteria were as follows: female patients who had undergone biopsy of the breast lumps, had a confirmed diagnosis of breast cancer, and had reports available for histopathology, as well as the receptor status, were included. Patients without available histopathology reports or histopathology reports lacking receptor status information were excluded from the study.

Information was gathered on socio-demographic factors such as age, gender, histopathological diagnosis of the patients, grade and stage of carcinoma, receptor status of ER, PR, HER2/NEU, and ki-67 marker status. The receptor status was further divided into subtypes as triple positive, triple negative, ER positive/PR negative/HER2/NEU positive, PR positive/ER negative/HER2/NEU negative, HER2NEU negative/ER positive/PR positive, ER negative/PR negative/HER2/NEU positive and ER positive/PR negative/HER2/NEU negative.

HER2/NEU 0 and 1 scores were taken as negative receptor status. In patients having a HER2/NEU score of 2, the Fluorescence In Situ Hybridization (FISH) test was conducted. If FISH was negative, then receptor status was counted as negative, but if the FISH test was positive, then receptor status was marked as positive. A HER2/NEU score of 3 was taken as receptor-positive. To evaluate the clinical validity of Ki-67, we used cut-offs as recommended by the International Ki-67 Breast Cancer Working Group (IKWG). Ki-67 ≤ 5% was considered low proliferation index, Ki-67 6% to 29% intermediate, and Ki-67 ≥ 30% as high proliferation index.

All data were analyzed using the Statistical Package for Social Sciences (SPSS) version 26 (IBM Corp., Armonk, USA). Categorical variables such as gender, grade, and stage of carcinoma status were presented as frequencies and percentages, while continuous variables such as age were presented as mean and standard deviation. The association between age and receptor status type was explored using the chi-square test. Statistical significance was set at a p-value of < 0.05. 

## Results

A total of 1003 patients with breast cancer were included in this study. All 1003 patients included were females. The mean age of the patients was 50.5 ± 11.83 years, with a range from 23 to 83 years. The patients were divided into the following age groups: 20-30 years (4.6%), 31-40 years (16.2%), 41-50 years (29.9%), 51-60 years (29.5%), and over 60 years (19.8%). 

**Figure 1:**
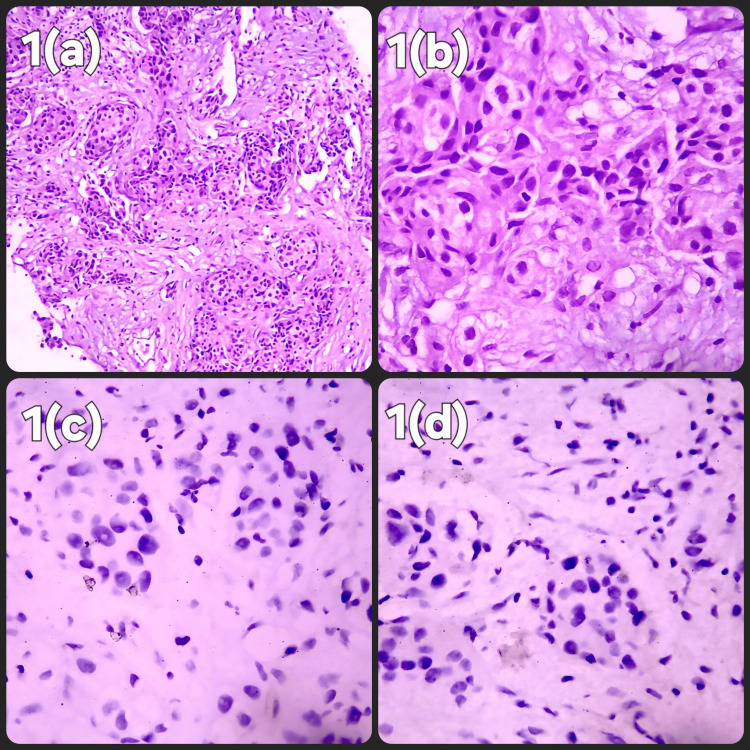
Histopathological and Immunohistochemical Features of Invasive Ductal Carcinoma (a) Invasive ductal carcinoma, Nottingham Grade 2, hematoxylin and eosin (H&E) stain, low-power field (LPF). (b) Invasive ductal carcinoma, Nottingham Grade 2, H&E stain, high-power field (HPF). (c) Estrogen receptor (ER) immunohistochemistry (IHC) stain shows negative nuclear stain - Allred score 0. (The Allred score accounts for the proportion of nuclear staining and staining intensity. A score of 0 indicates negative staining, while scores from 1 to 8 qualify as positive staining with increasing intensity of receptor positivity.) (d) Progesterone receptor (PR) IHC stain shows negative nuclear stain - Allred score 0.

**Figure 2:**
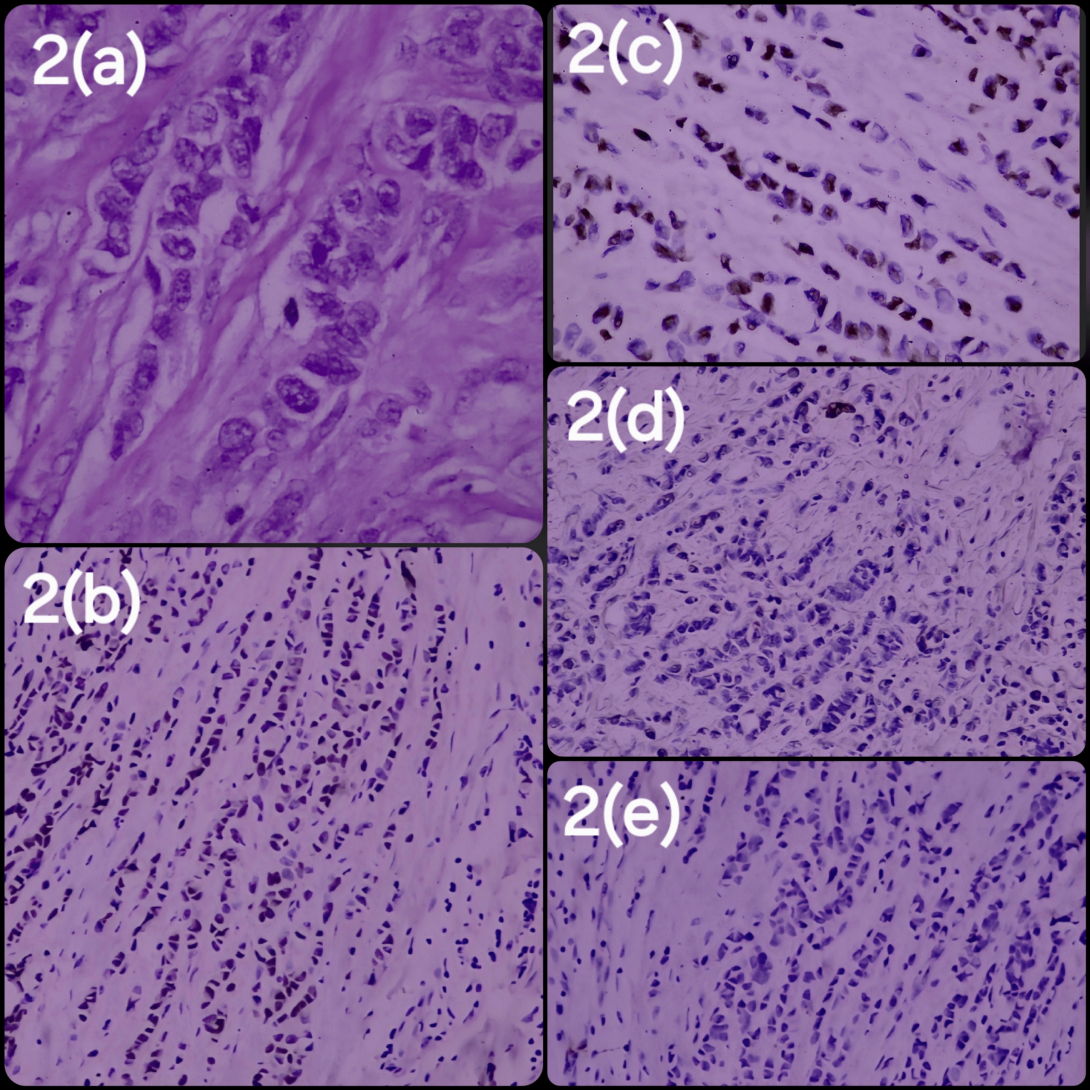
Histopathological and Immunohistochemical Profile of Invasive Lobular Carcinoma (a) Invasive lobular carcinoma, Nottingham Grade 1, hematoxylin and eosin (H&E) stain, high-power field (HPF); (b) estrogen receptor (ER) positive immunohistochemistry (IHC) nuclear stain, Allred score 8; (c) progesterone receptor (PR) nuclear stain, IHC positive, Allred score 7; (d) human epidermal growth factor receptor 2 (HER2) negative (score 0) IHC stain; (e) E-cadherin loss on IHC stain, diagnostic of invasive lobular carcinoma.

Most patients (91.0%) were diagnosed with invasive ductal carcinoma (IDC). The remaining histopathological diagnoses were invasive lobar carcinoma (ILC) (7.4%), invasive micropapillary carcinoma (IMC) (1.2%), and mucinous carcinoma (0.4%). Most patients had Grade II carcinoma (50.6%), followed by Grade III (45.5%) and then Grade I (3.9%). The most common stage was III (36.8%) and II (34.3%), followed by stage IV (25.6%) and stage I (3.3%). ER status: 62.4% of the patients were ER-positive, while 37.6% were ER-negative. PR status: 52.3% of the patients were PR-positive and 47.7% were PR-negative. HER2/NEU status: 44.0% were HER2/NEU-positive, and 56.0% were HER2/NEU-negative. Ki-67 marker testing was performed in 416 patients (41.5%). Among those tested, 0.9% had a Ki-67 level equals or <5%, 18.7% had a Ki-67 level between 6% and 29%, and 21.8% had a Ki-67 level equal to or >30% (Figures 1, 2). 

Grade II carcinoma was most common in patients in the age range of 20-30 years (54.3%), 51-60 years (57.1%), and over 60 years (54.3%). Grade III carcinoma was predominant in the patients of 31-40 years (59.3%) and 41-50 years (49.0%) age groups. The difference in the distribution of carcinoma grades among age groups was statistically significant (p < 0.05). Stage II and IV carcinomas were most common in the 20-30 years age group (34.8% each). Stage III carcinoma was most frequent in patients aged 31-40 years (43.8%), 41-50 years (40.3%), and 51-60 years (36.5%). In the age group of over 60 years, Stage II was the most common (42.7%). The difference between the age groups was not statistically significant (p = 0.05) (Tables 1, 2 ). 

**Table 1:** Stages of Carcinoma

Age Groups	Stage (n (%))				χ² (df=12)	P-value
	I	II	III	IV		
20-30 years	0 (0.0%)	16 (34.8%)	14 (30.4%)	16 (34.8%)	20.99	0.05
31-40 years	4 (2.5%)	54 (33.3%)	71 (43.8%)	33 (20.4%)		
41-50 years	8 (2.7%)	95 (31.7%)	121 (40.3%)	76 (25.3%)		
51-60 years	12 (4.1%)	94 (31.8%)	108 (36.5%)	82 (27.7%)		
>60 years	9 (4.5%)	85 (42.7%)	55 (27.6%)	50 (25.1%)		

**Table 2:** Grades of Carcinoma

Age Groups	Grade (n (%))			χ² (df=8)	P-value
	I	II	III		
20-30 years	0 (0.0%)	25 (54.3%)	21 (45.7%)	40.36	0.00
31-40 years	6 (3.7%)	60 (37.0%)	96 (59.3%)		
41-50 years	7 (2.3%)	146 (48.7%)	147 (49%)		
51-60 years	8 (2.7%)	169 (57.1%)	119 (40.2%)		
>60 years	18 (9%)	108 (54.3%)	73 (36.7%)		

ER positivity was highest in the 20-30 years age group (71.7%) and lowest in the 41-50 years age group (58.0%). The distribution of ER status among age groups was not statistically significant (p = 0.310). PR positivity was the highest in the 20-30 years age group (60.9%) and the lowest in the 51-60 years age group (48.3%). The distribution of PR status among age groups was also not statistically significant (p = 0.219). HER2/NEU positivity was the highest in the 41-50 years age group (48.7%) and the lowest in the 31-40 years age group (37.7%). The distribution of HER2/NEU status among age groups was not statistically significant (p = 0.17).

IDC was the most common histopathological type in all age groups. The distribution was as follows: 93.5% in the 20-30 years, 92.6% in the 31-40 years, 92.3% in the 41-50 years, 90.2% in the 51-60 years, and 88.4% in the over 60 years age group. The distribution of Ki-67 levels varied among age groups. In the 20-30 years age group, 26.1% had Ki-67 levels between 6-29% and equal to or greater than 30%. In the 31-40 years age group, 24.1% had Ki-67 levels equal to or greater than 30%. In the 41-50 years age group, 25.0% had Ki-67 levels equal to or greater than 30%. In the 51-60 years age group, 19.6% had Ki-67 levels between 6-29%. In patients over 60 years of age, 19.1% had Ki-67 levels between 6-29%. These differences were not statistically significant (p = 0.177)

Among the patients, 20.9% were triple-positive, 17.6% were triple-negative, 28.9% were HER2/NEU-negative but ER- and PR-positive, and 11.9% were HER2/NEU-positive but ER- and PR-negative. The most common receptor status among all age groups was HER2/NEU negative, ER, and PR positive, with the following distribution: 43.5% in the 20-30 years, 34.0% in the 31-40 years, 25.7% in the 41-50 years, 27.0% in the 51-60 years, and 29.1% in the over 60 years age group. The distribution of receptor status among age groups was statistically significant (p < 0.000) (Table 3).

**Table 3:** Receptor Status Among Different Age Groups (n (%))

Age group	Triple positive	Triple negative	ER positive, PR negative, HER2/NEU positive	PR positive, ER negative, HER2/NEU negative	HER2/NEU negative, ER PR positive	HER2/NEU positive, ER PR negative	ER negative, PR negative, HER2//NEU positive	ER Negative, PR positive, HER2/NEU positive	ER positive, PR negative, HER2/NEU negative	χ² (df=32)	P-value
20-30 years	8 (17.4%)	5 (10.9%)	2 (4.3%)	0 (0%)	20 (43.5%)	3 (6.5%)	5 (10.9%)	0 (0%)	3 (6.5%)	73.12	0.0000
31-40 years	36 (22.2%)	39 (24.1%)	7 (4.3%)	3 (1.9%)	55 (34%)	9 (5.6%)	9 (5.6%)	0 (0%)	4 (2.5%)		
41-50 years	64 (21.3%)	49 (16.3%)	16 (5.3%)	11 (3.7%)	77 (25.7%)	24 (8%)	42 (14%)	0 (0)	17 (5.7%)		
51-60 years	54 (18.2%)	49 (16.9%)	16 (5.4%)	3 (1%)	80 (27%)	14 (4.7%)	34 (11.5%)	7 (2.4%)	39 (13.2%)		
> 60 years	48 (24.1%)	35 (17.6%)	8 (4%)	0 (0%)	58 (29.1%)	6 (3%)	29 (14.6%)	1 (0.5%)	14 (7%)		

The distribution of hormone receptor status among patients with breast cancer across different stages is summarized in Table 3. In Stage I, the majority (57.6%) of the patients had HER2/NEU-negative but ER- and PR-positive tumors. Triple-positive cases accounted for 18.2%, and 12.1% were HER2/NEU-positive, but ER- and PR-negative. No cases were observed for ER-positive, PR-negative, HER2/NEU-positive, PR-positive, ER-negative, and HER2/NEU-negative status. Stage II had a similar distribution, with 25% of patients having HER2/NEU-negative, ER-, and PR-positive tumors, 24.7% were triple-positive, and 18.6% were triple-negative. Other receptor statuses were less common, ranging from 0.5% to 9.9%. In Stage III, HER2/NEU negative, ER-, and PR positive remained the most frequent at 28.7%, followed by triple positive (20.3%), and triple negative (19%). The distribution of the other receptor statuses ranged from 0.5% to 13.3%. For Stage IV, 30.7% of patients were HER2/NEU-negative, ER- and PR-positive, 17.1% triple-positive, and 16.3% triple-negative. The remaining receptor statuses varied between 0.8% and 12.5%. There was no significant difference in the hormone receptor status distribution across the different cancer stages (p = 0.76) (Table 4).

**Table 4:** Relationship of Stage of Carcinoma Among the Different Age Groups (n (%))

Stage of carcinoma	Triple positive	Triple negative	ER positive, PR negative, HER2/NEU positive	PR positive, ER negative, HER2/NEU negative	HER2/NEU negative, ER PR positive	HER2/NEU positive, ER PR negative	ER negative, PR negative, HER2/NEU Positive	ER negative, PR positive, HER2/NEU positive	ER positive, PR negative, HER2/NEU negative	χ² (df=24)	P-value
I	6 (18.2%)	1 (3.%)	0 (0%)	0 (0%)	19 (57.6%)	0 (0%)	4 (12.1%)	0 (0.0%)	3 (9.1%)	34.35	0.76
II	85 (24.7%)	64 (18.6%)	17 (4.9%)	8 (2.3%)	86 (25%)	15 (4.4%	34 (9.9%)	4 (1.2%)	31 (9.0%)		
III	75 (20.3%)	70 (19.0%)	18 (4.9%)	6 (1.6%)	106 (28.7%)	21 (5.7%)	49 (13.3%)	2 (0.5%)	22 (6%)		
IV	44 (17.1%)	42 (16.3%)	14 (5.4%)	3 (1.2%)	79 (30.7%)	20 (7.8%)	32 (12.5%)	2 (0.8%)	21 (8.2%)		

## Discussion

This study involved 1003 patients diagnosed with breast cancer, with a mean age of 50.5 years. The age distribution highlights a concentration of cases in middle-aged patients, particularly between 41 and 60 years. This aligns with existing literature, indicating that breast cancer incidence increases with age, peaking around menopause and slightly decreasing thereafter [11]. Invasive ductal carcinoma (IDC) was the predominant histopathological subtype, accounting for 91% of the cases. This finding is consistent with numerous studies that have established IDC as the most common type of breast cancer, representing approximately 70% to 80% of all breast cancers [12-14].

Grade II carcinoma was the most prevalent (50.6%), followed by Grade III (45.5%) and Grade I (3.9%). The higher frequency of Grade II and III carcinomas indicates moderate to poorly differentiated tumors, which are known to have a higher likelihood of aggressive behavior and poorer prognosis compared to Grade I tumors [15]. The significant variation in carcinoma grades across different age groups suggests that younger patients are more likely to present with higher-grade tumors, particularly those in the 31 to 40 years age group, where Grade III was the most common, while older patients over the age of 60 years more frequently had Grade II tumors.

The majority of patients were ER-positive (62.4%) and PR-positive (52.3%), which is consistent with global data indicating that hormone receptor-positive breast cancers are more prevalent. HER2/NEU positivity was observed in 44% of patients, a figure slightly higher than the typically reported 20% to 30% in Western populations, suggesting possible regional variations in HER2/NEU expression or differences in testing criteria. Ki-67, a marker of cell proliferation, was tested in 41.5% of the patients. Higher Ki-67 levels (equal to or >30%) were observed in a substantial proportion of patients, particularly in younger age groups. This finding supports the notion that younger patients often present with more aggressive tumor biology, as indicated by higher proliferation indices [16].

The age-related variation in tumor grade is critical as it impacts treatment decisions and prognosis [17]. However, the stage of carcinoma did not show a significant difference across the age groups (p = 0.05), suggesting that while younger patients may present with higher-grade tumors, the stage at diagnosis may not necessarily differ. This could reflect effective screening and early detection efforts across all age groups [18]. The distribution of ER and PR statuses did not vary significantly across age groups, indicating that hormone receptor expression was relatively consistent regardless of age. However, the slight decrease in ER positivity in the 41 to 50 years age group warrants further investigation to understand the underlying biological factors. HER2/NEU positivity also did not differ significantly across age groups. Notably, the highest HER2/NEU positivity was in the 41 to 50 years age group. The consistent HER2/NEU expression across age groups emphasizes the importance of HER2-targeted therapies across the spectrum of breast cancer patients [19, 20].

The most common receptor status across all age groups was HER2/NEU negative and ER- and PR-positive, observed in 28.9% of the patients. The distribution of combined receptor statuses was significantly different among age groups (p < 0.000), indicating that younger patients had more aggressive triple-negative breast cancers, while older patients more frequently had hormone receptor-positive, HER2-negative cancers. Studies have shown that triple-negative breast cancers have the worst prognosis [21]. This highlights the necessity for age-specific treatment strategies to optimize outcomes.

An analysis of hormone receptor statuses across different stages of breast cancer revealed distinct patterns. In Stage I, the majority of tumors (57.6%) were HER2/NEU-negative, ER-positive, and PR-positive, indicating a generally favorable prognosis and good response to hormonal therapies. Triple-positive cases constituted 18.2% of cases, also suggesting a better prognosis due to the presence of ER and PR positivity alongside HER2/NEU positivity. In Stage II, the most common tumors were again HER2/NEU-negative, ER-positive, and PR-positive (25%). Triple-positive cases were prevalent at 24.7%, and triple-negative cases at 18.6%, indicating a more diverse receptor status distribution, which could imply varied treatment responses within this group. Stage III tumors maintained a similar trend, with HER2/NEU-negative, ER-, and PR-positive tumors being the most frequent (28.7%). The presence of triple-positive (20.3%) and triple-negative (19%) cases highlights the receptor status heterogeneity in more advanced stages. The relatively high percentage of triple-negative cases is particularly concerning due to their association with poorer prognosis and limited treatment options.

In Stage IV, 30.7% of the patients had HER2/NEU-negative, ER-, and PR-positive tumors. There was an increase in triple-negative cases (16.3%) and HER2/NEU-positive, ER-, and PR-negative tumors (12.5%), which are known for their aggressive nature and challenging treatment responses. The overall p-value of 0.76 indicates no significant difference in hormone receptor status distribution across cancer stages, suggesting consistency in receptor patterns regardless of disease stage. The consistent prevalence of HER2/NEU-negative, ER-, and PR-positive tumors emphasizes the importance of hormone receptor testing for guiding treatment decisions throughout the disease course.

These findings support the existing literature that younger women tend to present with more aggressive breast cancer types, such as triple-negative or HER2/NEU-positive tumors (5, 11, 21). The data underscore the critical role of hormone receptor status in determining prognosis and tailoring treatment strategies for breast cancer patients. Further research is needed to explore the mechanisms underlying these patterns and develop targeted therapies for different stages of breast cancer. Additionally, the regional variations in HER2/NEU positivity rates suggest that local population-specific studies are essential to understand the biological and environmental factors influencing these differences. This knowledge could lead to more effective screening, diagnosis, and treatment protocols tailored to the specific needs of different demographic groups.

Limitations

Single-center data collection, incomplete Ki-67 testing, and lack of follow-up data on patient outcomes limit the generalizability and comprehensiveness of the findings. Further research is needed to explore regional variations and other molecular markers in breast cancer biology.

## Conclusions

This study confirms that invasive ductal carcinoma remains the most common subtype of breast cancer, with significant variations in tumor grade and receptor status across different age groups. Younger patients tend to present with more aggressive disease, characterized by higher-grade tumors and higher Ki-67 indices. These findings emphasize the need for personalized treatment approaches based on age, tumor grade, and receptor status to improve prognosis and survival outcomes in breast cancer patients.
